# Taxonomic diversity and abundance of enchytraeids (Annelida, Clitellata, Enchytraeida) in the Northern Palaearctic. 2. European Russia

**DOI:** 10.3897/BDJ.13.e144992

**Published:** 2025-02-24

**Authors:** Ruslan A. Saifutdinov, Maxim I. Degtyarev, Daniil I. Korobushkin, Svetlana M. Artemieva, Philipp S. Byzov, Margarita A. Danilova, Alla A. Ditts, Ksenia A. Ermokhina, Petr G. Garibian, Anastasia Yu. Gorbunova, Polina A. Guseva, Evgeniy I. Karlik, Tatiana Yu. Kondratieva, Dmitri A. Kupriyanov, Iurii M. Lebedev, Natalia V. Lebedeva, Pavel A. Nazarov, Alisa A. Neplyukhina, Elizaveta A. Noskova, Roman R. Obolensky, Alexey A. Panchenkov, Anna V. Popova, Nina A. Pronina, Joel Rüthi, Gabriela Schaepman-Strub, Svetlana V. Shakhab, Andrey S. Zaitsev, Vitalii A. Zemlianskii, Elena Yu. Zvychaynaya, Konstantin B. Gongalsky

**Affiliations:** 1 A.N. Severtsov Institute of Ecology and Evolution, Russian Academy of Sciences, Moscow, Russia A.N. Severtsov Institute of Ecology and Evolution, Russian Academy of Sciences Moscow Russia; 2 Institute of Biology, Komi Scientific Centre of the Ural Branch, Russian Academy of Sciences, Syktyvkar, Russia Institute of Biology, Komi Scientific Centre of the Ural Branch, Russian Academy of Sciences Syktyvkar Russia; 3 Present affiliation: UCD School of Biology and Environmental Science, University College Dublin, Dublin, Ireland Present affiliation: UCD School of Biology and Environmental Science, University College Dublin Dublin Ireland; 4 Present affiliation: Independent Researcher, Unaffiliated, Moscow, Russia Present affiliation: Independent Researcher, Unaffiliated Moscow Russia; 5 Institute of Geography, Russian Academy of Sciences, Moscow, Russia Institute of Geography, Russian Academy of Sciences Moscow Russia; 6 Present affiliation: Department of Biomedical Sciences, City University of Hong Kong, Hong Kong, China Present affiliation: Department of Biomedical Sciences, City University of Hong Kong Hong Kong China; 7 Murmansk Marine Biological Institute, Russian Academy of Sciences, Murmansk, Russia Murmansk Marine Biological Institute, Russian Academy of Sciences Murmansk Russia; 8 A.N. Belozersky Institute Of Physico-Chemical Biology, Lomonosov Moscow State University, Moscow, Russia A.N. Belozersky Institute Of Physico-Chemical Biology, Lomonosov Moscow State University Moscow Russia; 9 Present affiliation: Centre for Strategic Planning of FMBA of Russia, Moscow, Russia Present affiliation: Centre for Strategic Planning of FMBA of Russia Moscow Russia; 10 Present affiliation: All-Russian Center for Plant Quarantine, Moscow, Russia Present affiliation: All-Russian Center for Plant Quarantine Moscow Russia; 11 Swiss Federal Institute for Forest, Snow and Landscape Research, Birmensdorf, Switzerland Swiss Federal Institute for Forest, Snow and Landscape Research Birmensdorf Switzerland; 12 Department of Evolutionary Biology and Environmental Studies, University of Zurich, Zürich, Switzerland Department of Evolutionary Biology and Environmental Studies, University of Zurich Zürich Switzerland; 13 Senckenberg Museum of Natural History Görlitz, Görlitz, Germany Senckenberg Museum of Natural History Görlitz Görlitz Germany

**Keywords:** sampling event, soil fauna, potworm, tundra, boreal forest, deciduous forest, steppe, desert, Russia, mesofauna, Enchytraeidae, terrestrial ecosystem, oligochaete, Caucasus Mountains, Novaya Zemlya Archipelago, Franz Josef Land

## Abstract

**Background:**

Enchytraeids, commonly known as potworms, are small oligochaetes found worldwide in various terrestrial, freshwater and marine ecosystems. Despite their crucial role in ecosystem functioning, the diversity and abundance of Enchytraeidae are seldom studied due to the labour-intensive process of species identification. This study aims to address this gap and expand knowledge on the distribution and abundance of enchytraeids within the Northern Palaearctic Region. The provided dataset represents the latest and most comprehensive field sampling of enchytraeid communities within the European part of Russia within the Northern Palaearctic. It consists exclusively of an original set of soil samples systematically collected across the region from 2019 to 2023, without any previously published data included.

**New information:**

The dataset includes occurrences from 204 georeferenced sites, encompassing 73 species from 17 genera, totalling 61,254 records, with 1,419 records having the "present'’ occurrence status. This comprehensive, species-specific dataset (Darwin Core Archive – DwC-A) provides insights into the distribution and abundance of terrestrial enchytraeids across a wide geographic area, covering the eastern sector of the East European Plain and the North Caucasus Region within the Northern Palaearctic. Compiled from field sampling campaigns, this dataset is essential for exploring and understanding local and regional enchytraeid diversity over time and space. It also serves as a valuable resource for monitoring and conserving soil biodiversity in the studied region.

## Introduction

Enchytraeids, commonly known as potworms, are relatively small oligochaete worms with a wordlwide distribution (Erséus et al. 2010, Rota and de Jong 2015). Being genotypically flexible, they inhabit various habitats and ecosystems, including soils, marine littoral zones, freshwater lakes and streams and even the snow of glaciers (Nurminen 1973, Petersen and Luxton 1982, Didden 1993). It is generally believed that in soil they act as saprophages (Potapov et al. 2022) and are categorised with respect to their preferred occurrence in the soil profile into litter dwellers, soil dwellers and intermediate species (Römbke et al. 2017). This classification was recently substantiated using stable isotopic analysis, distinguishing them into epigeic, epi-endogeic and endogeic secondary decomposers (Korobushkin et al. 2024).

There are currently about 820 species from 36 genera of enchytraeids known worldwide, with several new species being described annually (Rota and de Jong 2015, Schmelz and Collado 2015, Martin et al. 2024). Recent studies show that, in European Russia, there are approximately 74 species belonging to 15 genera that inhabit soil (Degtyarev et al. 2019, Degtyarev et al. 2024). However, due to the highly laborious nature of *in vivo* species identification and the lack of taxonomic experts, there is still insufficient knowledge regarding spatial and temporal (e.g. seasonal dynamics) distribution of this group within the European Plain, especially in its eastern part (Rota and de Jong 2015, Römbke et al. 2017, Vorobeichik et al. 2021).

To address this knowledge gap, we have conducted extensive soil sampling across various terrestrial ecosystems within the Northern Palaearctic. Previously, we presented a dataset dedicated to the enchytraeid communities in the Asiatic part of the Northern Palaearctic (Degtyarev et al. 2024). This article is the result of our continued research and provides a comprehensive dataset on the abundance and distribution of terrestrial enchytraeids collected from various biomes in the Russian part of the East European Plain and the Caucasus Region within the Northern Palaearctic.

## General description

### Purpose

The purpose of the current data paper is to expand our knowledge on the abundance, diversity and distribution of enchytraeid communities in the Northern Palaearctic Region, particularly in the European part of Russia.

## Project description

### Study area description

The area under investigation is the European part of the Northern Palaearctic, specifically European Russia. It encompasses a diverse range of biome types, including tundra, boreal forests, temperate forests, grasslands, wetlands and coastal areas. Our research primarily focuses on the East European Plain, although some sampling sites extend beyond this region to include the Northern Caucasus Mountains, Novaya Zemlya Archipelago and Franz Josef Land. Therefore, we limit our research area by Franz Josef Land to the north, the Caucasus Mountains to the south and the Ural Mountains to the east. In total, we examined 204 sites located within various biomes as classified by WWF (Olson et al. 2001), including:

1. tundra;
2. boreal forest;
3. temperate broadleaf and mixed forest;
4. temperate grassland, savannah and shrubland;
5. desert and xeric shrubland.

In each of the biomes, we sampled a different number of sites due to logistical constraints and various extraction capacities. Comprehensive information about each site is given in Table 1.

## Sampling methods

### Sampling description

The material for the dataset was collected between 2019 and 2023. We selected sampling sites in areas that were not heavily disturbed by human activity. In arid regions, we chose the most humid (yet not flooded) spots. The sampling protocol was developed, based on widely recognised methods (Ghilarov 1975, Coleman et al. 2004) and in accordance with international standards (ISO 23611-3 2019). At each site, we collected a random selection of 1-8 soil cores. These soil cores were taken using a 5-cm-diameter steel corer down to a depth of 10 cm. After collection, the soil was carefully placed into plastic bags and transported to the laboratory at the A.N. Severtsov Institute of Ecology and Evolution, Russian Academy of Sciences, Moscow. Subsequently, the soil samples were stored in a refrigerator at +4°C until extraction. Enchytraeids were extracted from the soil without heating, using the Graefe's wet funnel technique (Didden et al. 1995). We placed a sieve in each funnel and a soil sample in each sieve. Then tap water was poured into the funnel so that the soil was completely covered. A test tube was attached to each funnel and placed in a container with water at room temperature. This precaution aimed to prevent potential overheating of the extracted enchytraeids, considering the possibility of random and sudden temperature fluctuations in the extraction room. Extraction was carried out for 16 to 24 hours, after which the tubes were detached from the funnels and the contents of the tubes were poured into Petri dishes.

### Quality control

The samples were collected by a number of soil zoologists and ecologists from the A.N. Severtsov Institute of Ecology and Evolution, Russian Academy of Sciences, Moscow, colleagues from other scientific institutes and trained volunteers. In total, 73 different enchytraeid species were collected. Enchytraeid species were identified *in vivo* immediately after the extraction procedure, according to Schmelz and Collado (2010). For species not included in this guide or described later, comparisons with original descriptions were used.

A total of 747 soil samples were collected from 204 sites, resulting in 61,254 records, of which only 1,419 indicated a "present" status, while the remaining 59,836 were classified as "absent". These "absences" refer specifically to the soil samples collected within our project and should be interpreted with caution when extrapolating the distributions of certain species at larger spatial scales. Nevertheless, we believe that including data records with an "absent" status may be beneficial for future biogeographic and soil ecological research.

It is important to note that the dataset presented does not claim to provide a comprehensive and objective account of enchytraeid diversity in the studied area due to several limitations related to sampling and extraction constraints. Our focus was primarily on the most typical habitats, which may have resulted in the omission of certain species, particularly those associated with aquatic environments. Additionally, the number of soil samples and the depth at which they were collected in each habitat may not have been sufficient to capture the full range of species diversity.

Despite these limitations, the presence data for specific species remain ecologically and biogeographically significant. All samples were collected during the vegetation period (spring to autumn), deliberately avoiding particularly dry summer periods. The population dynamics of many enchytraeid species are still largely unknown; therefore, species presence data are considered more reliable and informative than abundance data, as the latter are specific to a particular timeframe. Finally, while the extraction method employed does not guarantee 100% efficiency, it is comparable in effectiveness to other commonly-used enchytraeid extraction techniques (Kobetičová and Schlaghamerský 2003).

The taxonomy follows the WoRMS database (Martin et al. 2024). Scientific names were checked using the GBIF species matching tool. Since most individuals were used for *in vivo* identification without permanent preparation, for subsequent isotopic analysis, all instances of enchytraeid occurrences within the studied sites were documented as dwc:basisOfRecord=HumanObservation. Juvenile specimens were identified at the genus level. The identification of all enchytraeids was conducted by Maxim Degtyarev.

### Step description

1. Selection of study sites, choosing undisturbed areas displaying minimal or no signs of human activity.
2. Site sampling was carried out at a distance of no less than 100 m from the borders of selected natural sites within one of the seven biome types according to the WWF map of biomes (Olson et al. 2001).
3. At each site, soil cores were collected using a steel corer with a diameter of 5 cm, reaching a depth of 10 cm.
4. The transportation of soil samples was conducted in isothermic containers to prevent soil overheating, which could lead to the mortality of organisms present.
5. Enchytraeids were extracted from the soil using the wet funnel method as described by Didden et al. (1995).
6. Following the extraction procedure, enchytraeids were identified *in vivo* to the species level using an Olympus BX-43 microscope. Subsequently, they were preserved in 96% alcohol for further isotopic analyses.
7. Identified individuals were counted to determine abundance within the 10 cm soil core and then extrapolated from a 5 cm diameter to 1 m². Extrapolation was performed by multiplying the number of individuals by a coefficient of 512 to account for sampling bias induced by the slight ellipsoidality of the corers, which arises from their production technology peculiarities. Given the variance in the number of soil samples across sites, the dataset includes abundance expressed as individuals per square metre.

## Geographic coverage

### Description

**Figure 1.**
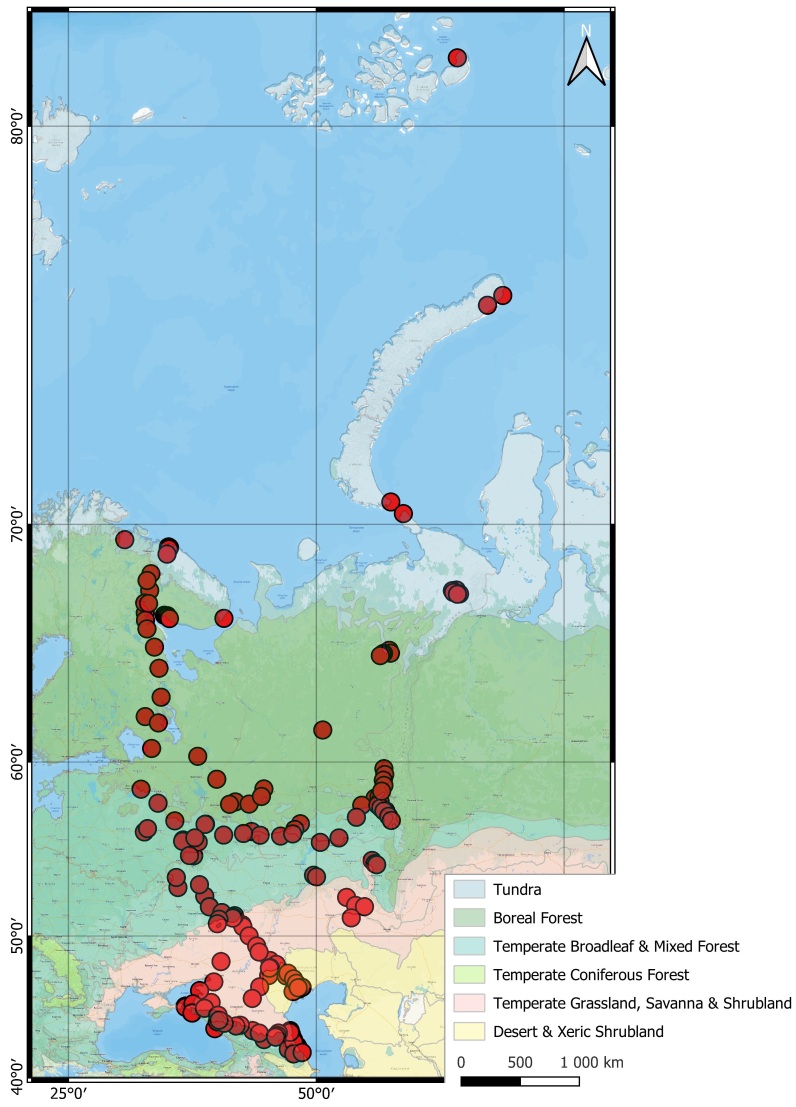
Enchytraeidae sampling locations in the Eastern European part of the Northern Palaearctic. Polygons in different colours illustrate biomes according to Olson et al. (2001). The map was created using QGIS 3.40.1 - Bratislava software (QGIS.org 2024). The map layer was created using Yandex Maps 4x with the WGS 84/Pseudo-Mercator projection. The shapefile with biomes was downloaded from www.ecoregions.appspot.com (Dinerstein et al. 2017).

The research area is located in the East European part of the Northern Palaearctic, with most sampling sites situated in the East European Plain (Fig. 1). Additionally, the research area includes the northern macroslope of the Caucasus Mountains to the south, as well as the Novaya Zemlya Archipelago and Franz Josef Land to the north. This extensive geographic area encompasses diverse habitat types, including tundra, boreal forest, steppe, deciduous forest and alpine habitats. Spanning a large latitudinal gradient, the region transitions from a tundra climate (ET) in the north to a subarctic climate (Dfc), warm-summer humid continental climate (Dfb), hot-summer humid continental climate (Dfa), humid subtropical climate (Cfa) and cold semi-arid climate (Bsk) in the south, according to the Köppen-Geiger climate classification (Rubel et al. 2017). Furthermore, in the mountainous regions of the Caucasus, the climate gradually changes from a hot-summer humid continental climate (Dfa) at lower elevations to a tundra climate (ET) at the peaks.

The geographical references were obtained by recording the coordinates of the sampling sites using a mobile phone and the Organic Maps app (Organic Maps 2024) or using Garmin GPSMAP 66s. The measurement error of the coordinates was approximately 25 m. The WGS84 coordinate system was used for all records.

### Coordinates

41.7411 and 81.129 Latitude; 30.676 and 68.8152 Longitude.

## Taxonomic coverage

### Description

Across the 204 sampling sites studied within five biomes in the European Russian part of the Northern Palaearctic, we identified a total of 73 species belonging to 17 genera (Table 2). The most frequently observed species were *Enchytraeusbuchholzi* s.l. (present in 79 out of 204 sites), *Cognettiasphagnetorum* s.l. (62 sites), *Buchholziaappendiculata* (48 sites) and *Fridericiabulboides* (46 sites).

Several species were identified with the "cf." designation, indicating that they were similar to, but not definitively identified as, those species (Table 2). Specimens of Fridericiacf.ulrikae were much smaller compared to the original description (5 mm vs. 13–18 mm in total length; 36–37 vs. 50–55 segments) and had five pairs of preclitellar nephridia instead of four. The shape of the Achaetacf.danica spermatheca more closely resembles that of the newly-described *A.florens*, but careful re-investigation is impossible due to the loss of specimens. Fridericiacf.ilvana had colourless blood instead of pink-yellow. In the case of Achaetacf.diddeni, specimens from Ingushetia were not fully mature, while specimens from Yaroslavl Oblast were significantly larger compared to the original description (6–7 mm vs. 4 mm in total length). Specimens of Marioninacf.magnaglandulosa were subadult (the spermatheca was not yet fully formed) and very small (1.5–2 mm long), possibly representing exceptionally small *M.filiformis*. For Henleacf.nasuta, yellowish epidermal gland cells were present, which is unusual for the genus. All specimens of Cernosvitoviellacf.atrata were subadult. In the case of Henleacf.montana, the quality of the obtained material does not allowed us to make a definitive conclusion.

The highest species richness was recorded in temperate broadleaf and mixed forests (73 species in total, see Table 3). Boreal forests hosted significantly fewer species; we found only 27. The number of species in the tundra and temperate grasslands, savannahs and shrublands varied between 17 and 21, respectively. The lowest species richness was observed in xeric shrublands and deserts (Table 3). The average species richness of enchytraeids varied between 5.4 species per site in temperate broadleaf and mixed forests and 0.3 species per site in deserts and xeric shrublands.

The highest average abundance of enchytraeids, reaching up to 13,000 individuals per square metre, was observed in temperate broadleaf and mixed forests (Table 3). A relatively lower density was found in boreal forests, with approximately 10,000 individuals per square metre, as well as in temperate grasslands, savannahs and shrublands, which had about 6,000 individuals. The lowest abundance was recorded in deserts and xeric shrublands, with only 1,000 individuals per square metre or fewer.

### Taxa included

**Table:** 

Rank	Scientific Name	Common Name
family	Enchytraeidae	potworm

## Usage licence

### Usage licence

Other

### IP rights notes

This work is licensed under a Creative Commons Attribution 4.0 International Licence (CC BY 4.0).

## Data resources

### Data package title

Taxonomic diversity and abundance of enchytraeids (Annelida, Clitellata, Enchytraeida) in the Northern Palaearctic. 2. European Russia

### Resource link

https://www.gbif.org/dataset/315256c2-842e-4da2-a7e0-72fe4f9ab671

### Alternative identifiers

https://doi.org/10.15468/74zp2f, doi.org/10.5281/zenodo.14257966

### Number of data sets

1

### Data set 1.

#### Data set name

Taxonomic diversity and abundance of enchytraeids (Annelida, Clitellata, Enchytraeida) in the Northern Palaearctic. 2. European Russia

#### Data format

Darwin Core format TXT file (tab delimited values)

#### Description

The description of each observation (occurrence) in the dataset follows the terms used in the general Darwin Core vocabulary (GBIF 2023). To optimally organise the dataset, we adhered to the recommendations proposed by Shashkov et al. (2021) with some modifications. The dataset represents a sampling-event dataset that includes a Darwin Core Event file and a Darwin Core Occurrence Extension. The Darwin Core Event file contains all necessary information about the sampling events. Since we collected many individual samples using a soil corer, we decided to present data obtained from a single soil core as a unique event (with a unique identifier in the eventID column). Consequently, all soil cores collected within a single sampling site were assigned a parentEventID, which serves as the unique identifier for the sampling site across the sampling area (the European part of the Northern Palaearctic). Each soil core (event) and, therefore, each sampling site (parentEvent) includes basic information, such as the coordinates of the sampling location, the date of sampling, the sampling protocol, the size of the sample and the biome to which the site belongs (in the "habitat" column). The Darwin Core Occurrence Extension file represents the occurrence of each species found in the research area as a unique occurrence within a unique event. Each occurrence includes information about the person who collected the soil core (recordedBy), whether the species is present or absent (occurrenceStatus), the person who identified the species (identifiedBy), the abundance of the species expressed as individuals per square metre and the full systematics of the species.

**Data set 1.** 

Column label	Column description
eventID (Event core, Occurrence extension)	Each event is assigned a unique identifier constructed from the sampling date, country code, region abbreviation, sampling site numbe, and the number of the soil core. For example, the identifier "2020-07-03-RU-TA-S58-SMP1" corresponds to soil core #1 (event) collected on 3 July 2020, at sampling site #58 in the Republic of Tatarstan.
parentEventID (Event core, Occurrence extension)	Each parentEventID serves as the identifier for the sampling site across the sampling area (the European part of the Northern Palaearctic) and may encompass several unique events. It consists of the sampling date, country code, region abbreviation, and sampling site number. For example, the identifier "2022-06-14-RU-BA-S177" corresponds to sampling site #177 in the Republic of Bashkortostan, where samples (events) were collected on 14 June 2022.
eventDate (Event core)	Date on which soil cores were collected, formatted as YYYY-MM-DD (year-month-day) according to ISO 8601.
day (Event core)	The integer day of the month on which the dwc:Event occurred.
month (Event core)	The integer month in which the dwc:Event occurred.
year (Event core)	The four-digit year in which the dwc:Event occurred, according to the Common Era Calendar.
samplingProtocol (Event core)	A constant value describing the extraction method used for all sampling events. The protocol was wet extraction of animals from 19.6 cm^2^ soil cores using funnels.
samplingEffort (Event core)	The number of soil samples collected and processed using the extraction procedure.
sampleSizeValue (Event core)	A numeric value for a measurement of the size (volume) of a sample in a sampling dwc:Event.
sampleSizeUnit (Event core)	The unit of measurement of the size (volume) of a sample in a sampling dwc:Event.
decimalLatitude (Event core)	The geographic latitude (in decimal degrees, using the spatial reference system given in dwc:geodeticDatum) of the geographic centre of a dcterms:Location.
decimalLongitude (Event core)	The geographic longitude (in decimal degrees, using the spatial reference system given in dwc:geodeticDatum) of the geographic centre of a dcterms:Location.
coordinatePrecision (Event core)	A decimal representation of the precision of the coordinates given in the dwc:decimalLatitude and dwc:decimalLongitude.
coordinateUncertaintyInMetres (Event core)	The horizontal distance (in metres) from the given dwc:decimalLatitude and dwc:decimalLongitude describing the smallest circle containing the whole of the dcterms:Location.
geodeticDatum (Event core)	The ellipsoid, geodetic datum or spatial reference system (SRS), upon which the geographic coordinates given in dwc:decimalLatitude and dwc:decimalLongitude are based. Constant value - "WGS84".
country (Event core)	The name of the country or major administrative unit in which the dcterms:Location occurs.
countryCode (Event core)	The standard code for the country in which the dcterms:Location occurs.
stateProvince (Event core)	The name of the next smaller administrative region than country (state, province, canton, department, region etc.), in which the dcterms:Location occurs. For sampling events, this records the federal subject (republic, krai, oblast etc.) where the sample was collected.
habitat (Event core)	This variable provides the biome classification assigned to the sampling location, based on the habitat typing system defined by the World Wildlife Fund (WWF). For additional information about the WWF biome classification system, please refer to Olson et al. (2001).
type (Event core)	The nature or genre of the resource. Constant value - event.
occurrenceID (Occurrence extension)	Each occurrence is assigned a unique identifier constructed from the sampling date, country code, region abbreviation for Russia, sampling site number, soil core number and occurrence number within that soil core. For example, the identifier "2021-10-20-RU-YR-S164-SMP1-21" corresponds to the 21^st^ occurrence recorded in soil core #1 on 20 October 2021, at sampling site #164 in Yaroslavl Oblast, Russia.
basisOfRecord (Occurrence extension)	This field contains a constant value indicating the record type. All occurrences have the value "Human observation" because organisms were identified *in vivo* and then used for further isotopic analysis after collection.
recordedBy (Occurrence extension)	The person, group or organisation responsible for originally recording the occurrence data. For example: "Degtyarev M | Gongalsky K".
identifiedBy (Occurrence extension)	The person, group or organisation responsible for identification. For all records in this dataset, organisms were identified by Maxim Degtyarev.
organismQuantity (Occurrence extension)	A number or enumeration value for the quantity of dwc:Organisms.
organismQuantityType (Occurrence extension)	The type of quantification system used for the quantity of dwc:Organisms.
occurrenceStatus (Occurrence extension)	A statement about the presence or absence of a dwc:Taxon at a dcterms:Location.
scientificName (Occurrence extension)	The full scientific name, with authorship and date information, if known. Example: "Marioninamagnaglandulosa Nurminen, 1970".
identificationQualifier (Occurrence extension)	Column for standard term (e.g. "cf."') used with species name to indicate uncertainty about the dwc:Identification.
identificationRemarks (Occurrence extension)	Freeform remarks entered relevant to the taxonomy and characterisation of the documented species or taxon. Example: "Marionina juveniles".
kingdom (Occurrence extension)	The full scientific name of the kingdom in which the dwc:Taxon is classified.
phylum (Occurrence extension)	The full scientific name of the phylum or division in which the dwc:Taxon is classified.
class (Occurrence extension)	The full scientific name of the class in which the dwc:Taxon is classified.
order (Occurrence extension)	The full scientific name of the order in which the dwc:Taxon is classified.
family (Occurrence extension)	The full scientific name of the family in which the dwc:Taxon is classified.
genus (Occurrence extension)	The full scientific name of the genus in which the dwc:Taxon is classified.
scientificNameAuthorship (Occurrence extension)	The authorship information for the dwc:scientificName formatted according to the conventions of the applicable dwc:nomenclaturalCode.
taxonRank (Occurrence extension)	The taxonomic rank of the most specific name in the dwc:scientificName.

## Figures and Tables

**Table 1.** Locations, habitat information and the number of recorded enchytraeid species for sampling sites in the European Northern Palaearctic Region. Unidentified enchytraeids were excluded from species counts and juvenile specimens were included only if no species from the same genus were present at the site. The classification of biomes is given according to Olson et al. (2001).

Biome	Site ID	Vegetation / Biotope	Species Recorded
Tundra	2019-09-20-RU-MU-S48	Hypoarctic tundra	0
Tundra	2020-08-08-RU-MU-S75	Hypoarctic tundra	3
Tundra	2020-08-08-RU-MU-S76	Hypoarctic tundra	5
Tundra	2020-08-08-RU-MU-S77	Hypoarctic tundra	6
Tundra	2020-08-08-RU-MU-S78	Hypoarctic tundra	4
Tundra	2020-08-08-RU-MU-S79	Hypoarctic tundra	5
Tundra	2020-08-09-RU-MU-S80	Hypoarctic tundra	3
Tundra	2020-08-09-RU-MU-S81	Hypoarctic tundra	3
Tundra	2020-08-09-RU-MU-S82	Hypoarctic tundra	2
Tundra	2020-08-10-RU-MU-S83	Hypoarctic tundra	2
Tundra	2020-08-10-RU-MU-S84	Hypoarctic tundra	1
Tundra	2020-08-11-RU-MU-S90	Hypoarctic tundra	1
Tundra	2021-06-15-RU-AR-S147	Polar desert	2
Tundra	2022-07-15-RU-AR-S191	Polar desert with some mosses and lichens	3
Tundra	2022-07-15-RU-AR-S192	Polar desert with some mosses and lichens	4
Tundra	2022-08-03-RU-AR-S193	Arctic tundra	3
Tundra	2022-08-04-RU-AR-S194	Arctic tundra	4
Tundra	2023-07-21-RU-KO-S200	Hypoarctic tundra	4
Tundra	2023-07-21-RU-KO-S201	Hypoarctic tundra	3
Tundra	2023-07-21-RU-KO-S202	Hypoarctic tundra	3
Tundra	2023-07-21-RU-KO-S203	Hypoarctic tundra	3
Tundra	2023-07-21-RU-KO-S204	Hypoarctic tundra	2
Boreal Forest	2019-07-13-RU-PM-S39	Southern taiga	5
Boreal Forest	2019-09-17-RU-MU-S44	Northern taiga	1
Boreal Forest	2019-09-17-RU-MU-S45	Northern taiga	4
Boreal Forest	2019-09-17-RU-MU-S46	Swampy Pinus forest	2
Boreal Forest	2019-09-20-RU-KL-S49	Tundra with mosses and prostrate shrubs	1
Boreal Forest	2019-09-20-RU-MU-S47	Pinus forest with Picea	1
Boreal Forest	2020-07-03-RU-MR-S53	Picea - Betula forest	4
Boreal Forest	2020-07-04-RU-PM-S64	Betula - Picea forest	7
Boreal Forest	2020-07-05-RU-KO-S68	Picea forest	2
Boreal Forest	2020-08-01-RU-LO-S69	Pinus forest with Vaccinium myrtillus	1
Boreal Forest	2020-08-01-RU-LO-S70	Pinus forest with Vaccinium and Erica	1
Boreal Forest	2020-08-02-RU-KL-S74	Populus tremula - Pinus forest with Sorbus, various cereals and forbs	7
Boreal Forest	2020-08-02-RU-MU-S73	Northern taiga	1
Boreal Forest	2020-08-10-RU-KL-S87	Picea - Pinus forest with Betula and Vaccinium myrtillus	1
Boreal Forest	2020-08-10-RU-KL-S88	Pinus forest with Vaccinium myrtillus	0
Boreal Forest	2020-08-10-RU-KL-S89	Swampy Pinus forest with Vaccinium myrtillus	1
Boreal Forest	2020-08-10-RU-MU-S85	Betula - Pinus forest with Ledum and Vaccinium myrtillus	1
Boreal Forest	2020-08-10-RU-MU-S86	Pinus forest with Betula, Populus tremula and Vaccinium myrtillus	2
Boreal Forest	2020-08-12-RU-MU-S91	Northern taiga	1
Boreal Forest	2020-08-12-RU-MU-S92	Northern taiga	2
Boreal Forest	2020-08-12-RU-MU-S93	Northern taiga	6
Boreal Forest	2020-08-12-RU-MU-S94	Northern taiga	4
Boreal Forest	2020-08-12-RU-MU-S95	Northern taiga	2
Boreal Forest	2020-08-12-RU-MU-S96	Northern taiga	3
Boreal Forest	2020-08-12-RU-MU-S97	Northern taiga	5
Boreal Forest	2020-08-12-RU-MU-S98	Northern taiga	4
Boreal Forest	2020-08-16-RU-KL-S100	Populus tremula - Pinus forest	1
Boreal Forest	2020-08-16-RU-KL-S99	Mixed grove near the lake	3
Boreal Forest	2020-08-16-RU-VO-S101	Taiga forest with some deciduous undergrowth	6
Boreal Forest	2020-08-16-RU-VO-S102	Mixed grove	2
Boreal Forest	2020-08-30-RU-KS-S106	Pinus - Picea coniferous forest	1
Boreal Forest	2020-08-31-RU-KS-S108	Betula - Picea forest with rare grass	3
Boreal Forest	2020-08-31-RU-KS-S109	Betula - Picea forest with Vaccinium myrtillus	4
Boreal Forest	2021-06-30-RU-KS-S148	Picea - Pinus forest	1
Boreal Forest	2021-06-30-RU-KS-S149	Pinus forest with Betula and Vaccinium myrtillus	1
Boreal Forest	2021-06-30-RU-KS-S150	Pinus forest with Betula and Vaccinium myrtillus	1
Boreal Forest	2022-06-20-RU-PM-S186	Picea - Pinus forest with Vaccinium myrtillus and mosses	1
Boreal Forest	2022-06-20-RU-PM-S187	Betula - Picea forest with Sorbus and ferns	4
Boreal Forest	2022-06-20-RU-PM-S188	Populus tremula - Picea forest with ferns	4
Boreal Forest	2022-06-20-RU-PM-S189	Picea forest with ferns	3
Boreal Forest	2022-06-20-RU-PM-S190	Populus tremula - Picea forest	4
Boreal Forest	2023-07-19-RU-KO-S195	Southern tundra	1
Boreal Forest	2023-07-19-RU-KO-S196	Southern tundra	1
Boreal Forest	2023-07-19-RU-KO-S197	Southern tundra	2
Boreal Forest	2023-07-19-RU-KO-S198	Southern tundra	1
Boreal Forest	2023-07-19-RU-KO-S199	Southern tundra	1
Temp. Broadleaf and Mixed Forest	2019-04-27-RU-MA-S1	Fraxinus - Acer forest with Quercus	11
Temp. Broadleaf and Mixed Forest	2019-04-27-RU-MA-S2	Betula - Acer forest	11
Temp. Broadleaf and Mixed Forest	2019-05-08-RU-LP-S17	Ulmus - Quercus forest with Festuca and Urtica	7
Temp. Broadleaf and Mixed Forest	2019-05-08-RU-TL-S18	Fraxinus - Quercus forest with Festuca	11
Temp. Broadleaf and Mixed Forest	2019-05-08-RU-VR-S19	Quercus forest with Pinus	3
Temp. Broadleaf and Mixed Forest	2019-05-08-RU-VR-S20	Acer forest with Corylus and Asarum	7
Temp. Broadleaf and Mixed Forest	2019-05-09-RU-VR-S21	Broadleaf grove	5
Temp. Broadleaf and Mixed Forest	2019-05-10-RU-VR-S22	Quercus - Acer forest with Corylus and Lysimachia	4
Temp. Broadleaf and Mixed Forest	2019-05-10-RU-VR-S23	Quercus forest with Crataegus and Convallaria majalis	8
Temp. Broadleaf and Mixed Forest	2019-05-10-RU-VR-S24	Quercus forest with Corylus	5
Temp. Broadleaf and Mixed Forest	2019-05-10-RU-VR-S25	Deciduous grove	1
Temp. Broadleaf and Mixed Forest	2019-06-04-RU-YR-S26	Broadleaf - Picea forest with forbs and Apiaceae	3
Temp. Broadleaf and Mixed Forest	2019-06-17-RU-KR-S27	Quercus and Carpinus orientalis forest	11
Temp. Broadleaf and Mixed Forest	2019-06-18-RU-KR-S28	Quercus and Carpinus orientalis forest	7
Temp. Broadleaf and Mixed Forest	2019-06-19-RU-KR-S29	Broadleaf forest with predominance of Quercus, Fagus and Carpinus betulus; with Sambucus, Corylus, Polygonatum, Viola, Hedera	4
Temp. Broadleaf and Mixed Forest	2019-07-13-RU-PM-S37	Mixed forest	15
Temp. Broadleaf and Mixed Forest	2019-07-13-RU-PM-S38	Southern taiga	6
Temp. Broadleaf and Mixed Forest	2019-07-15-RU-OR-S40	Quercus forest	7
Temp. Broadleaf and Mixed Forest	2019-08-16-RU-MO-S41	Populus tremula - Picea forest	10
Temp. Broadleaf and Mixed Forest	2019-08-16-RU-MO-S42	Picea forest with Populus tremula and Sorbus	4
Temp. Broadleaf and Mixed Forest	2019-09-05-RU-KR-S43	Subtropical deciduous forest	6
Temp. Broadleaf and Mixed Forest	2019-10-15-RU-DA-S51	Deciduous liana forest with predominance of Ulmus and Quercus	4
Temp. Broadleaf and Mixed Forest	2020-07-03-RU-CU-S52	Acer forest with Quercus undergrowth	6
Temp. Broadleaf and Mixed Forest	2020-07-03-RU-MR-S54	Pinus forest with Betula, Calamagrostis and ferns	2
Temp. Broadleaf and Mixed Forest	2020-07-03-RU-MR-S55	Abies - Tilia forest with Acer undergrowth	3
Temp. Broadleaf and Mixed Forest	2020-07-03-RU-NN-S56	Pinus forest with Betula	1
Temp. Broadleaf and Mixed Forest	2020-07-03-RU-NN-S57	Quercus forest	6
Temp. Broadleaf and Mixed Forest	2020-07-03-RU-TA-S58	Deciduous forest	3
Temp. Broadleaf and Mixed Forest	2020-07-03-RU-TA-S59	Deciduous forest	3
Temp. Broadleaf and Mixed Forest	2020-07-03-RU-VL-S60	Betula - Quercus forest with forbs	3
Temp. Broadleaf and Mixed Forest	2020-07-03-RU-VL-S61	Mixed forest with Pinus, Picea and Betula	2
Temp. Broadleaf and Mixed Forest	2020-07-04-RU-PM-S62	Southern taiga	4
Temp. Broadleaf and Mixed Forest	2020-07-04-RU-PM-S63	Southern taiga with deciduous undergrowth	4
Temp. Broadleaf and Mixed Forest	2020-07-04-RU-PM-S65	Deciduous grove	5
Temp. Broadleaf and Mixed Forest	2020-07-04-RU-PM-S66	Picea - Betula forest	5
Temp. Broadleaf and Mixed Forest	2020-07-04-RU-UD-S67	Picea - Pinus forest with Betula	4
Temp. Broadleaf and Mixed Forest	2020-08-01-RU-TV-S71	Picea - Betula forest	10
Temp. Broadleaf and Mixed Forest	2020-08-01-RU-TV-S72	Pinus - Picea - Betula forest	8
Temp. Broadleaf and Mixed Forest	2020-08-29-RU-MO-S103	Picea - Betula forest with Sorbus, Carex and Aegopodium	5
Temp. Broadleaf and Mixed Forest	2020-08-29-RU-MO-S104	Mixed forest	8
Temp. Broadleaf and Mixed Forest	2020-08-29-RU-TV-S105	Betula - Picea forest with Corylus and Oxalis	5
Temp. Broadleaf and Mixed Forest	2020-08-30-RU-TV-S107	Picea forest with Acer, ferns and forbs	3
Temp. Broadleaf and Mixed Forest	2020-09-01-RU-SR-S110	Steppe meadow with rare Tilia	5
Temp. Broadleaf and Mixed Forest	2020-09-01-RU-SR-S111	Steppe meadow	6
Temp. Broadleaf and Mixed Forest	2020-09-15-RU-KG-S112	Deciduous forest with predominance of Tilia, Fraxinus and Quercus; with Corylus, Carex, Aegopodium and Asarum	5
Temp. Broadleaf and Mixed Forest	2020-10-01-RU-KR-S115	Deciduous forest with predominance of Carpinus betulus, Quercus and Carpinus orientalis	4
Temp. Broadleaf and Mixed Forest	2020-10-10-RU-AD-S117	Quercus - Ulmus forest	3
Temp. Broadleaf and Mixed Forest	2020-10-11-RU-KC-S119	Ulmus forest with Urtica	8
Temp. Broadleaf and Mixed Forest	2020-10-11-RU-KC-S120	Deciduous forest with predominance of Ulmus, Fraxinus and Acer; with Rosa, Euonymus and Viburnum	5
Temp. Broadleaf and Mixed Forest	2020-10-12-RU-IN-S121	Pinus - Quercus - Acer mixed forest	5
Temp. Broadleaf and Mixed Forest	2020-10-13-RU-DA-S123	Broadleaf forest with predominance of Carpinus betulus and Fagus	4
Temp. Broadleaf and Mixed Forest	2020-10-14-RU-DA-S129	Dry cereal meadow	0
Temp. Broadleaf and Mixed Forest	2020-10-14-RU-DA-S130	Cereal steppe with Tamarix	0
Temp. Broadleaf and Mixed Forest	2020-10-15-RU-DA-S131	Deciduous liana forest with predominance of Quercus and Populus	5
Temp. Broadleaf and Mixed Forest	2020-10-15-RU-DA-S132	Deciduous liana forest with predominance of Quercus and Populus, local Iberis thickets	8
Temp. Broadleaf and Mixed Forest	2020-10-15-RU-DA-S133	Deciduous liana forest	4
Temp. Broadleaf and Mixed Forest	2020-10-15-RU-DA-S134	Deciduous liana forest	5
Temp. Broadleaf and Mixed Forest	2020-10-15-RU-DA-S135	Broadleaf forest	4
Temp. Broadleaf and Mixed Forest	2020-10-16-RU-DA-S140	Broadleaf forest	6
Temp. Broadleaf and Mixed Forest	2021-04-21-RU-AD-S144	Quercus forest	10
Temp. Broadleaf and Mixed Forest	2021-04-21-RU-AD-S145	Floodplain Tilia - Alnus forest	9
Temp. Broadleaf and Mixed Forest	2021-04-21-RU-KR-S141	Fagus forest	10
Temp. Broadleaf and Mixed Forest	2021-04-21-RU-KR-S142	Fagus - Abies forest	13
Temp. Broadleaf and Mixed Forest	2021-04-21-RU-KR-S143	Fagus forest	3
Temp. Broadleaf and Mixed Forest	2021-09-25-RU-MO-S151	Pinus forest with Betula, Vaccinium myrtillus and Oxalis	2
Temp. Broadleaf and Mixed Forest	2021-10-10-RU-MO-S152	Pinus forest with Vaccinium myrtillus, Sorbus aucuparia and Sphagnum	5
Temp. Broadleaf and Mixed Forest	2021-10-15-RU-DA-S155	Broadleaf forest	6
Temp. Broadleaf and Mixed Forest	2021-10-15-RU-DA-S156	Deciduous forest	4
Temp. Broadleaf and Mixed Forest	2021-10-18-RU-CN-S160	Quercus forest with Crataegus	0
Temp. Broadleaf and Mixed Forest	2021-10-18-RU-CN-S161	Fagus - Carpinus betulus forest with Corylus	3
Temp. Broadleaf and Mixed Forest	2021-10-18-RU-CN-S162	Deciduous forest	1
Temp. Broadleaf and Mixed Forest	2021-10-18-RU-CN-S163	Alnus forest	3
Temp. Broadleaf and Mixed Forest	2021-10-20-RU-YR-S164	Tilia - Pinus forest with ferns	4
Temp. Broadleaf and Mixed Forest	2022-04-25-RU-DA-S175	Deciduous liana forest	2
Temp. Broadleaf and Mixed Forest	2022-06-07-RU-MO-S176	Picea and Acer forest with Oxalis, Sorbus and Convallaria	4
Temp. Broadleaf and Mixed Forest	2022-06-14-RU-BA-S177	Deciduous forest	3
Temp. Broadleaf and Mixed Forest	2022-06-14-RU-BA-S178	Deciduous forest	6
Temp. Broadleaf and Mixed Forest	2022-06-14-RU-BA-S179	Deciduous forest	3
Temp. Broadleaf and Mixed Forest	2022-06-14-RU-BA-S180	Deciduous forest	11
Temp. Broadleaf and Mixed Forest	2022-06-18-RU-PM-S181	Picea - Betula forest with Sorbus and ferns	5
Temp. Broadleaf and Mixed Forest	2022-06-18-RU-PM-S182	Mixed forest with Picea, Populus tremula, Betula and ferns	3
Temp. Broadleaf and Mixed Forest	2022-06-18-RU-PM-S183	Betula forest with Picea and Sorbus	6
Temp. Broadleaf and Mixed Forest	2022-06-18-RU-PM-S184	Picea - Betula forest with Pinus	9
Temp. Broadleaf and Mixed Forest	2022-06-18-RU-PM-S185	Pinus - Picea - Betula forest	8
Temp. Grassland, Savannah and Shrubland	2019-04-29-RU-AO-S3	Steppe with Artemisia and Stipa	0
Temp. Grassland, Savannah and Shrubland	2019-04-29-RU-AO-S4	Steppe with Artemisia and Agropyron	0
Temp. Grassland, Savannah and Shrubland	2019-05-02-RU-KM-S9	Semi-desert with Artemisia and Bromus	0
Temp. Grassland, Savannah and Shrubland	2019-05-03-RU-KM-S10	Semi-desert with Artemisia, Bromus and Elytrigia	0
Temp. Grassland, Savannah and Shrubland	2019-05-03-RU-KM-S11	Semi-desert with Tamarix and Salsola	0
Temp. Grassland, Savannah and Shrubland	2019-05-04-RU-VG-S12	Forb - cereal steppe	5
Temp. Grassland, Savannah and Shrubland	2019-05-04-RU-VG-S13	Forb - cereal steppe	3
Temp. Grassland, Savannah and Shrubland	2019-05-04-RU-VG-S14	Forb - cereal steppe	8
Temp. Grassland, Savannah and Shrubland	2019-05-04-RU-VG-S15	Forb - cereal steppe	8
Temp. Grassland, Savannah and Shrubland	2019-05-04-RU-VG-S16	Steppe with Artemisia and Stipa	0
Temp. Grassland, Savannah and Shrubland	2019-06-24-RU-KR-S30	Steppe with Agropyron, Elytrigia and forbs	0
Temp. Grassland, Savannah and Shrubland	2019-06-24-RU-KR-S31	Steppe with Elytrigia, Agropyron and forbs	3
Temp. Grassland, Savannah and Shrubland	2019-06-26-RU-KR-S32	Steppe with predominance of Alopecurus, with Centaurea and Achillea	1
Temp. Grassland, Savannah and Shrubland	2019-06-26-RU-KR-S33	Steppe with predominance of Alopecurus, with Rumex and Centaurea	2
Temp. Grassland, Savannah and Shrubland	2019-06-26-RU-RO-S34	Cereal - forb - Artemisia steppe	0
Temp. Grassland, Savannah and Shrubland	2019-06-27-RU-RO-S35	Cereal - forb steppe with Artemisia	2
Temp. Grassland, Savannah and Shrubland	2019-06-27-RU-VR-S36	Cereal steppe with calciophylic plants	0
Temp. Grassland, Savannah and Shrubland	2019-10-14-RU-DA-S50	Broadleaf forest	2
Temp. Grassland, Savannah and Shrubland	2020-09-29-RU-KR-S113	Cereal - forb steppe	0
Temp. Grassland, Savannah and Shrubland	2020-09-29-RU-KR-S114	Steppe	0
Temp. Grassland, Savannah and Shrubland	2020-10-02-RU-KR-S116	Meadow steppe with few Malus trees	3
Temp. Grassland, Savannah and Shrubland	2020-10-11-RU-KB-S118	Quercus - Ulmus forest with Acer undergrowth	2
Temp. Grassland, Savannah and Shrubland	2020-10-12-RU-SO-S122	Ulmus - Acer deciduous forest with Corylus	3
Temp. Grassland, Savannah and Shrubland	2020-10-14-RU-DA-S124	Broadleaf forest	3
Temp. Grassland, Savannah and Shrubland	2020-10-14-RU-DA-S125	Deciduous grove	5
Temp. Grassland, Savannah and Shrubland	2020-10-14-RU-DA-S126	Dry steppe with Elaeagnus and Tamarix	0
Temp. Grassland, Savannah and Shrubland	2020-10-14-RU-DA-S127	Dry steppe with shrubs and cereals	0
Temp. Grassland, Savannah and Shrubland	2020-10-14-RU-DA-S128	Forb - cereal steppe	0
Temp. Grassland, Savannah and Shrubland	2020-10-16-RU-DA-S136	Cereal meadow with Tamarix	1
Temp. Grassland, Savannah and Shrubland	2020-10-16-RU-DA-S137	Desert steppe with Elaeagnus and Tamarix	0
Temp. Grassland, Savannah and Shrubland	2020-10-16-RU-DA-S138	Cereal steppe with Tamarix	0
Temp. Grassland, Savannah and Shrubland	2020-10-16-RU-DA-S139	Deciduous forest with predominance of Ulmus and Quercus	1
Temp. Grassland, Savannah and Shrubland	2021-04-29-RU-ST-S146	Forb steppe	1
Temp. Grassland, Savannah and Shrubland	2021-10-14-RU-DA-S153	Broadleaf forest	1
Temp. Grassland, Savannah and Shrubland	2021-10-14-RU-DA-S154	Dry steppe with Tamarix	0
Temp. Grassland, Savannah and Shrubland	2021-10-16-RU-DA-S157	Desert steppe	0
Temp. Grassland, Savannah and Shrubland	2021-10-16-RU-DA-S158	Desert steppe	0
Temp. Grassland, Savannah and Shrubland	2021-10-16-RU-DA-S159	Semi-desert	0
Temp. Grassland, Savannah and Shrubland	2022-04-23-RU-OB-S165	Forb - cereal steppe	6
Temp. Grassland, Savannah and Shrubland	2022-04-23-RU-OB-S166	Forb - cereal steppe	4
Temp. Grassland, Savannah and Shrubland	2022-04-23-RU-OB-S167	Forb - cereal steppe	6
Temp. Grassland, Savannah and Shrubland	2022-04-23-RU-OB-S168	Forb - cereal steppe	1
Desert and Xeric Shrubland	2019-04-29-RU-AO-S5	Floodplain Carex meadow	1
Desert and Xeric Shrubland	2019-04-30-RU-AO-S6	Stipa steppe	0
Desert and Xeric Shrubland	2019-04-30-RU-KM-S7	Semi-desert with Elytrigia and Papaver	0
Desert and Xeric Shrubland	2019-05-02-RU-KM-S8	Semi-desert with Tamarix, Elytrigia and Galium	0
Desert and Xeric Shrubland	2022-04-25-RU-AO-S169	Semi-desert	0
Desert and Xeric Shrubland	2022-04-25-RU-AO-S170	Semi-desert	0
Desert and Xeric Shrubland	2022-04-25-RU-AO-S171	Semi-desert	0
Desert and Xeric Shrubland	2022-04-25-RU-AO-S172	Semi-desert	0
Desert and Xeric Shrubland	2022-04-25-RU-AO-S173	Steppe near the river	1
Desert and Xeric Shrubland	2022-04-25-RU-AO-S174	Semi-desert	1

**Table 2.** List of enchytraeid taxa found in studied sites (n = number of sites where taxon is present) across biomes classified by WWF (Olson et al. 2001): Tundra (T), Boreal Forest (BF), Temperate Broadleaf and Mixed Forest (TBMF), Temperate Grassland, Savannah and Shrubland (TGS), Desert and Xeric Shrubland (DXS). The "comment" column provides additional information about the identification of specific taxa: cf. (confer) indicates that the specimen is similar to the listed species, but not definitively identified as such; s.l. (sensu lato) indicates that the classification is used in a broad sense, encompassing related or cryptic species.

Taxon	N	Comment	T	BF	TBMF	TGS	DXS
*Achaetaaffinis* Nielsen & Christensen, 1959	1				+		
*Achaetabibulba* Graefe, 1989	5				+		
*Achaetabohemica* (Vejdovský, 1879)	2				+		
*Achaetacamerani* (Cognetti, 1899)	2				+		
*Achaetadanica* Nielsen & Christensen, 1959	1	cf.			+		
*Achaetadiddeni* Graefe, 2007	2	cf.			+		
*Achaetaeiseni* Vejdovský, 1878	7				+		
*Achaetapannonica* Graefe, 1989	6			+	+		
*Achaetaunibulba* Graefe, Dózsa-Farkas & Christensen, 2005	2				+		
*Achaeta* sp. Vejdovský, 1878	1	juveniles			+		
*Bryodrilusparvus* Nurminen, 1970	2		+	+			
*Bryodrilus* sp. Ude, 1892	1	juveniles			+		
*Buchholziaappendiculata* (Buchholz, 1863)	48			+	+	+	
*Buchholziasimplex* Nielsen & Christensen, 1963	2				+		
*Cernosvitoviellaatrata* (Bretscher, 1903)	4	cf.	+	+			
*Cognettiabisetosa* Christensen & Dózsa-Farkas, 1999	5		+	+			
*Cognettiaglandulosa* (Michaelsen, 1888)	19	s.l.	+	+	+		
*Cognettialapponica* Nurminen, 1965	17		+	+	+		
*Cognettiasphagnetorum* (Vejdovský, 1878)	62	s.l.	+	+	+	+	
*Enchytraeusalbidus* Henle, 1837	1				+		
*Enchytraeusbuchholzi* Vejdovský, 1878	79	s.l.	+	+	+	+	
*Enchytraeusbulbosus* Nielsen & Christensen, 1963	1				+		
*Enchytraeuschristenseni* Dózsa-Farkas, 1992	1					+	
*Enchytraeuscoronatus* Nielsen & Christensen, 1959	1				+		
*Enchytraeusdichaetus* Schmelz & Collado, 2010	10				+	+	+
*Enchytraeuslacteus* Nielsen & Christensen, 1961	3				+	+	
*Enchytraeusnorvegicus* Abrahamsen, 1969	7				+	+	
*Enchytraeus* sp. Henle, 1837	6	juveniles			+	+	
*Enchytroniachristenseni* Dózsa-Farkas, 1970	1				+		
*Enchytroniaparva* Nielsen & Christensen, 1959	33			+	+	+	
*Fridericiaargillae* Schmelz, 2003	2				+	+	
*Fridericiabenti* Schmelz, 2002	10			+	+	+	
*Fridericiabisetosa* (Levinsen, 1884)	33			+	+	+	
*Fridericiabulboides* Nielsen & Christensen, 1959	46		+	+	+	+	
*Fridericiacallosa* (Eisen, 1878)	3		+				
*Fridericiachristeri* Rota & Healy, 1999	18				+	+	
*Fridericiaconnata* Bretscher, 1902	2				+		
*Fridericiacusanica* Schmelz, 2003	1				+		
*Fridericiacylindrica* Springett, 1971	3			+	+		
*Fridericiagalba* (Hoffmeister, 1843)	12				+	+	
*Fridericiaglobuligera* Rota, 1995	1				+		
*Fridericiagongalskyi* Degtyarev, 2023	2				+		
*Fridericiailvana* Issel, 1905	1	cf.			+		
*Fridericiaisseli* Rota, 1994	11				+	+	
*Fridericialacii* Dózsa-Farkas, 2009	6			+	+		
*Fridericialarix* Schmelz & Collado, 2005	2				+		
*Fridericiamaculata* Issel, 1905	14			+	+	+	
*Fridericiamaculatiformis* Dózsa-Farkas, 1972	1				+		
*Fridericianemoralis* Nurminen, 1970	3				+		
*Fridericiaparathalassia* Schmelz, 2003	1				+		
*Fridericiaparoniana* Issel, 1904	16				+	+	
*Fridericiaperrieri* (Vejdovský, 1878)	5			+	+		
*Fridericiaratzeli* (Eisen, 1872)	30			+	+	+	
*Fridericiasamurai* Degtyarev, 2022	5				+		
*Fridericiaschmelzi* Cech & Dózsa-Farkas, 2005	7			+	+		
*Fridericiasylvatica* Healy, 1979	3				+		
*Fridericiatuberosa* Rota, 1995	2				+		
*Fridericiaulrikae* Rota & Healy, 1999	2	cf.			+		
*Fridericia* sp. Michaelsen, 1889	40	juveniles		+	+	+	
*Globulidrilusriparius* (Bretscher, 1899)	2			+	+		
*Hemifridericiaparva* Nielsen & Christensen, 1959	3				+		
*Henleaglandulifera* Nurminen, 1970	1				+		
*Henleaheleotropha* Stephenson, 1922	2		+				
*Henleajutlandica* Nielsen & Christensen, 1959	1				+		
*Henleamontana* Rota, 1994	1	cf.			+		
*Henleanasuta* (Eisen, 1878)	3	cf.	+		+		
*Henleaperpusilla* Friend, 1911	17		+		+	+	
*Henleaventriculosa* (d'Udekem, 1854)	25			+	+	+	
*Henlea* sp. Michaelsen, 1889	9	juveniles	+		+	+	
*Lumbricillus* sp. Ørsted, 1844	1	juveniles	+				
*Marioninaargentea* (Michaelsen, 1889)	3	s.l.	+		+		
*Marioninacommunis* Nielsen & Christensen, 1959	5		+		+		
*Marioninafiliformis* Nielsen & Christensen, 1959	5			+	+		
*Marioninamagnaglandulosa* Nurminen, 1970	2	cf.		+	+		
*Marioninavesiculata* Nielsen & Christensen, 1959	4				+		
*Marionina* sp. Michaelsen, 1890	1	juveniles			+		
*Mesenchytraeusarmatus* (Levinsen, 1884)	2			+	+		
*Mesenchytraeuspelicensis* Issel, 1905	2				+		
*Mesenchytraeus* sp. Eisen, 1878	7	juveniles	+	+			
*Oconnorellacambrensis* (O'Connor, 1963)	1				+		
*Stercutusniveus* Michaelsen, 1888	18			+	+		
Enchytraeidae sp.	3	unidentified	+		+		

**Table 3.** Average species richness per sampling site within a biome (species per site ± SE), total species richness and average abundance (indiv. per square metre ± SE) of enchytraeids in the studied biomes of the European Russian part of the Northern Palaearctic. The numbers in brackets adjacent to specific biomes indicate the number of sampling sites. During the calculation of average and total species richness, unidentified enchytraeids were excluded. Additionally, juvenile specimens were included in the counts only if no species from the same genus were present at the same site. The classification of biomes is given according to Olson et al. (2001).

Biome	Average No of species	Total No of species	Average abundance, ind. m^2^
Tundra (n = 22)	3 ± 0.3	17	6317 ± 956
Boreal Forest (n = 46)	2.5 ± 0.3	27	9965 ± 1674
Temperate Broadleaf and Mixed Forest (n = 84)	5.4 ± 0.3	73	13242 ± 1649
Temperate Grassland, Savannah and Shrubland (n = 42)	1.7 ± 0.4	21	7847 ± 2109
Desert and Xeric Shrubland (n = 10)	0.3 ± 0.2	1	1248 ± 804
